# Fungicides modify pest insect fitness depending on their genotype and population

**DOI:** 10.1038/s41598-023-44838-5

**Published:** 2023-10-19

**Authors:** Aigi Margus, Shahed Saifullah, Maaria Kankare, Leena Lindström

**Affiliations:** Department of Biological and Environmental Science, University of Jyväskylä, P.O. Box 35, 40014 Jyväskylä, Finland

**Keywords:** Molecular biology, Ecology, Ecology, Agroecology, Evolutionary ecology, Invasive species, Evolution, Experimental evolution

## Abstract

Fungicides are the most sold pesticide group, with an 8% increase in sales in Europe within the last decade. While adverse short-term fungicide effects on non-target insect species have been reported, the long-term effects and their impact on fitness are unclear. As the effects may depend on both the fungicide and the genetic background of the species, we investigated the effects of the commonly used fungicide, fluazinam, on the Colorado potato beetle's life history traits, and whether the effects were dependent on a previously characterized insecticide resistance mutation (S291G in *acetylcholinesterase-2* gene) in different populations. Our findings show that fungicide exposure can have both negative and positive, long-lasting effects on beetles, depending on the parental insecticide resistance status and population. In the Belchow population, individuals carrying resistance mutation had higher survival, but they produced offspring with lower egg-hatching rates. While, in the Vermont population, fungicide exposure increased the body mass and offspring quality in the beetles carrying resistance mutation but did not affect the beetles’ survival. Our results suggest that commonly used fungicides can have both negative and positive effects on pest insects’ life-history, however, their impact may differ depending on the population and parental genetic background.

## Introduction

Fungicides were the most sold group of agricultural pesticides (based on mass) in the European Union (EU) in 2020, and the amounts of fungicides sold annually in the EU have increased by 8% since 2011^1^. Moreover, the globalisation of trade and environmental changes have intensified fungal disease dispersal^2^ and simultaneously increased fungicide use. Fungicides are used to prevent fungal pathogen damage in all major crops^3^ and even organic farming depends on fungicide application^4^. Unlike other pesticides, fungicides are typically applied to target crops up to 10 times per season^5^. The increase in usage and the need for multiple applications have resulted in a growing concern about fungicide's effects on biota beyond fungi and calls for more studies^6^.

Fungicide effects extend beyond the target fungi, as there are reports on the adverse effects on non-target species are species that live in the crop fields^7–11^. Studies using field-realistic concentrations of fungicides have reported toxic effects to several organisms, including beneficial pollinators, pest insects of the crops, as well as fish and other aquatic species in the lakes or rivers nearby the agricultural regions^6,12,13^. Reported fungicide effects on non-target insect species can also be more subtle and sublethal effects have been reported for example in the Japanese beetle (*Popillia japonica* Newman*;* reduced hatching of eggs and increased larval mortality)^14^*,* and in the Colorado potato beetle (*Leptinotarsa decemlineata* Say; delayed larval development)^15,16^. Studies have also suggested that exposure to fungicide (mancozeb) does rather manifest in later life stages, as delayed pupation and pupal mortality like in turnip moths (*Agrotis segetum* Denis & Schiffermüller)^17^. Moreover, exposed female turnip moths laid fewer eggs, which had some malformations (envelopes of nuclei were invaginated and swollen) suggesting that fungicide effects can carry over generations. In addition to effects on life-history traits, exposure to low concentrations of fungicides increased disease infections in bees, such as the Nosema caused by microsporidian parasites^18^ or increased the toxicity of the insecticides for bees^19^.

Not all non-target individuals are affected the same way by fungicides because of the variation among populations' genetic profiles^20^ and the genetic background of the individuals^10,15^. In agroecosystems, pest insects have been selected by insecticides, and resistance to these chemicals has resulted in many differences^21^, for example at the physiology (e.g. enzymatic activities) between resistant and susceptible individuals^22^. Therefore, individuals resistant to insecticides might be better at resisting also adverse fungicide effects if their ability to detoxify chemicals is higher in general. There is only one study which has directly compared how resistant and susceptible pest insect populations respond to fungicides^16^. In that study, fungicides were found to reduce larval survival and to delay larval development, but the effects were not related to insecticide resistance^16^.

**Figure 1:**
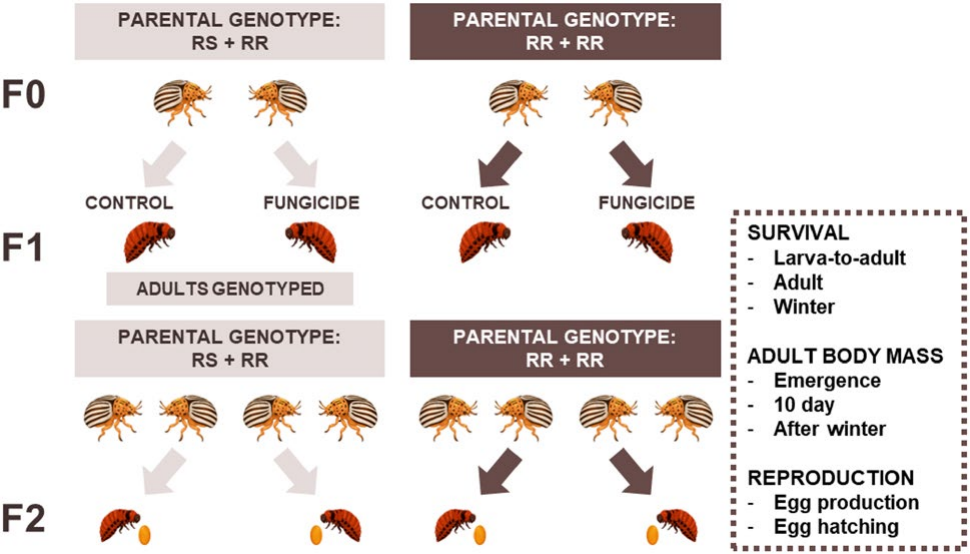
Experimental setup for testing how fungicide exposure affects the Colorado potato beetles depending on their parental resistance status and population (not shown on the figure). We genotyped and mated the individuals in the F0 generation, thereafter we mated the beetles according to their insecticide resistance status (*i.e.,* S291G mutation in the *acetylcholinesterase-2* gene). Beetles were either heterozygous (RS) or homozygous (RR) for that mutation. Their offspring (F1) were exposed to either control or fungicide treatment and thereafter, we measured their survival, body mass and reproduction (*i.e.,* production of the F2 generation). Beetle images are drawn by Janna Ratavaara.

**Figure 2:**
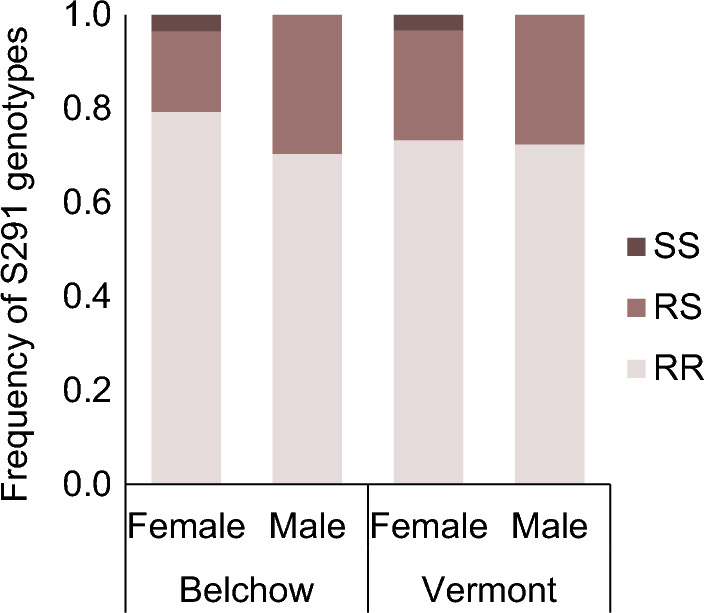
Frequency of genotypes for the organophosphate resistance associated mutation S291G in the *acetylcholinesterase-2* gene in the female and male Colorado potato beetles in two populations (Belchow and Vermont). SS—homozygous susceptible, lacking the mutation, RS—heterozygous, RR—homozygous for the organophosphate resistance-associated mutation.

We investigated within- and transgenerational effects of larval exposure to a commonly used fungicide (fluazinam, product name Shirlan) on the Colorado potato beetle (*Leptinotarsa decemlineata*) that is a common pest in agroecosystems. The mode of action of fluazinam targets the respiration process in fungi and it is categorised by FRAC as an uncoupler of oxidative phosphorylation^23^. This fungicide is used repeatedly against the late potato blight and thus the Colorado potato beetle is likely a non-target species for the fungicide. To investigate this further, we used two-by-two factorial design, where we aimed to assess how exposure to the fungicide and parental insecticide resistance background affect the beetle’s fitness and survival (Fig. 1). First, we studied the direct effects of the fungicide on several fitness components, including short and long-term survival, adult body mass, and reproduction in two beetle populations (Vermont, US and Belchow, Poland). Both populations have the same resistant mutation (S291) in the *acetylcholinesterase-2* gene that has been associated with insecticide resistance to carbamate and organophosphate insecticides^24,25^. Although the prevalence of this mutation is similar between the two populations (Fig. 2), the Vermont population survives 107-times higher concentrations of organophosphate and 20-times higher concentrations of carbamate insecticides than the Belchow population^22^ which could be either due to insecticide usage history or lower genetic variability among the European populations^26^. Second, we tested whether insecticide resistance mutation affects the responses to the fungicides within the population. In other words, are homozygous-resistant (RR) individuals less resistant to fungicide exposure than heterozygous (RS) individuals (see materials and methods)? We genotyped the adult beetles for S291G mutation in the *acetylcholinesterase-2* gene^27^ (Fig. 2) and hypothesize, based on earlier studies, that exposure to fungicide will have adverse effects on beetles life-history than those not exposed but the effects of fungicides should no be dependent on the insecticide background of the individuals^16^.

## Results

### Fungicide and parental genotype effects on survival

**Figure 3:**
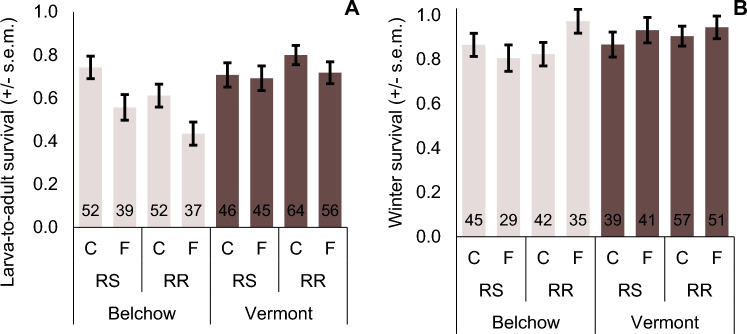
(**A**) Larva-to-adult and (**B**) winter survival (+ / − s.e.m.) in Belchow and Vermont populations. Fungicide exposure significantly reduces larva-to-adult survival in the Belchow population. RS marks the heterozygous and RR homozygous families for the S291G point mutation in the *acetylcholinesterase-2* gene. C marks for control and F for fluazinam fungicide treatment. Numbers in the column base show the sample size of the survived individuals.

Fluazinam-based fungicide exposure reduced larva-to-adult survival by 18% in the Belchow population compared to the control group, while there were no significant effects observed in the Vermont population (Table 1; Fig. 3A). This suggests that individuals from the Belchow population are more sensitive to fungicide exposure than individuals from the Vermont population. Furthermore, in the Belchow population, parents with resistance mutation produced offspring with 13% lower larva-to-adult survival than parents with mixed genotypes (Table 1, Fig. 3A). Fungicide exposure did not affect before diapause survival, but it had an interaction effect with parental genotype on the winter survival (= overwintering) (Table 1, Fig. 3B). Specifically, individuals from RR parents had 15% higher winter survival, and individuals from RS parents had 6% lower survival when exposed to fungicide than the control group in the Belchow population (Table 1, Fig. 3B). In contrast, in the Vermont population fungicide exposure, parental genotype or their interaction did not affect larva-to-adult, before diapause, or winter survival significantly (Table 1, Fig. 3).

**Table 1:** Fungicide exposure, parental insecticide resistance genotype (RR/RS), and their interaction affect the survival (larva-to-adult, within 10 days before diapause, and winter survival) of the Colorado potato beetle, from Belchow and Vermont populations.

Population	Model	Larva-to-adult survival		Before diapause survival		Winter survival	
		Wald χ2	p	Wald χ2	p	Wald χ2	p
Belchow	parental genotype	**5.22**	**0.022**	0	0.999	2.18	0.139
	fungicide exposure	**10.40**	**0.001**	0	0.999	1.65	0.199
	parental genotype* fungicide exposure	0.06	0.808	0	0.999	**4.00**	**0.046**
Vermont	parental genotype	1.36	0.244	< 0.01	0.936	0.33	0.566
	fungicide exposure	0.96	0.328	0.21	0.646	1.62	0.204
	parental genotype* fungicide exposure	0.50	0.480	0.19	0.663	0.02	0.877

For statistics, parents were either homozygous (RR) or heterozygous (RS) for the S291G mutation in the *acetylcholinesterase-2* gene. Significant results (p < 0.05) are shown in bold.

### Fungicide and parental genotype effects on the body mass

**Figure 4:**
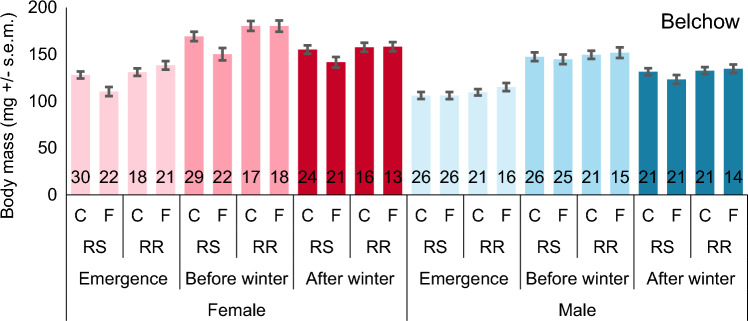
Body mass at different stages (mg + / s.e.m; emergence, before winter, and after winter) for female (red) and male (blue) beetles in Belchow population. RS marks the mixed and RR resistant families. C marks for control and F for fluazinam fungicide treatment. Numbers in the column base show the sample sizes.

Fungicide exposure and parental genotype affected the female emergence and overwintering adult body mass in the Belchow population but these differences disappeared over winter (Table 2, Fig. 4). Specifically, female beetles that descended from RR parents and were exposed to fungicide had higher emergence body mass (+ 7 mg) than those from the control group. In contrast, females descending from the RS parents exposed to fungicide had lower emergence body mass (− 18 mg) than those from the control group (Table 2; Fig. 4). However, this fungicide-parental genotype interaction effect disappeared before diapause in the Belchow population. Nonetheless, the parental genotype effect persisted, with females from RR parents having higher before winter mass (+ 21 mg) than females from the RS parents (Table 2; Fig. 4), though these positive effects disappeared again after winter diapause. In addition, we found no significant fungicide exposure, parental genotype, or their interaction effects on male body mass in the Belchow population (Table 2; Fig. 4).

**Table 2:** Fungicide exposure, parental insecticide resistance genotype (RS/RR), and their interaction affect the female and male body mass (emergence, before winter, and after winter) of the Colorado potato beetles, from Belchow and Vermont populations.

Population	Sex	Model	Emergence mass		Before winter mass		After winter mass	
			F_df_	p	F_df_	p	F_df_	p
Belchow	♀	parental genotype	**12.8**_**1,90**_	** < 0.001**	**12.6**_**1,88**_	** < 0.001**	3.6_1,77_	0.062
		fungicide exposure	1.5_1,90_	0.228	2.8_1,88_	0.099	1.7_1,77_	0.194
		parental genotype* fungicide exposure	**8.3**_**1,90**_	**0.005**	2.6_1,88_	0.110	2.1_1,77_	0.156
		family	1.7_1,90_	0.199	0.3_1,88_	0.569	1.3_1,77_	0.257
	♂	parental genotype	2.6_1,80_	0.108	0.9_1,75_	0.354	2.2_1,64_	0.146
		fungicide exposure	0.5_1,80_	0.471	< 0.01_1,75_	0.963	0.5_1,64_	0.461
		parental genotype* fungicide exposure	1.1_1,80_	0.466	0.3_1,75_	0.613	1.4_1,64_	0.237
		family	1.0_1,80_	0.324	0.8_1,75_	0.361	3.7_1,64_	0.059
Vermont	♀	parental genotype	**5.0**_**1,100**_	**0.027**	**14.9**_**1,99**_	** < 0.001**	**9.3**_**1,91**_	**0.003**
		fungicide exposure	0.4_1,100_	0.530	1.1_1,99_	0.288	0.2_1,91_	0.676
		parental genotype* fungicide exposure	**4.6**_**1,100**_	**0.034**	**7.2**_**1,99**_	**0.008**	**8.6**_**1,91**_	**0.004**
		family	0.3_1,100_	0.615	**9.0**_**1,99**_	**0.003**	2.7_1,91_	0.101
	♂	parental genotype	**6.6**_**1,101**_	**0.011**	1.0_1,95_	0.308	**4.8**_**1,87**_	**0.031**
		fungicide exposure	**12.1**_**1,101**_	** < 0.001**	**10.2**_**1,95**_	**0.002**	**7.8**_**1,87**_	**0.006**
		parental genotype* fungicide exposure	1.1_1,101_	0.287	0.2_1,95_	0.636	0.08_1,87_	0.774
		family	0.2_1,101_	0.659	0.7_1,95_	0.411	**4.0**_**1,87**_	**0.048**

Parents were either homozygous (RR) or heterozygous (RS) for the S291G mutation in the *acetylcholinesterase*-*2* gene. Significant results (p < 0.05) are shown in bold.

**Figure 5:**
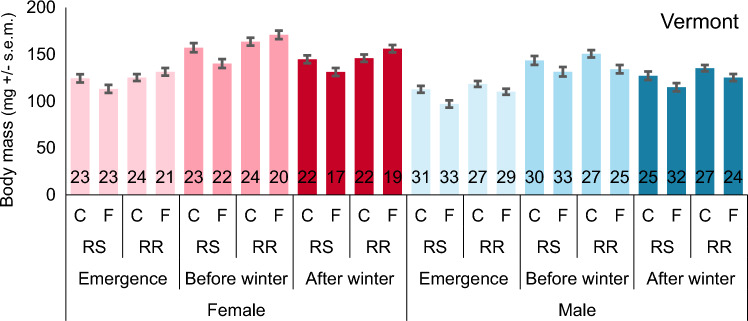
Body mass at different stages (+ / − s.e.m.: emergence, before winter, and after winter) for female (red) and male (blue) beetles in Vermont population. RS marks the mixed and RR resistant families. C marks for control group and F marks for fluazinam fungicide group. Numbers in the column base show the sample sizes.

In the Vermont population, we observed that fungicide exposure and parental genotype had an interactive effect on female body mass that remained from emerged adults until diapause termination (after winter-diapause; Table 2, Fig. 5), suggesting that fungicide effects are long-term and could be carried over to the next generation. For example, females from RR parents exposed to fungicide had, throughout, a slightly higher body mass (+ 6 mg) than those from the control group. In comparison, females from RS parents exposed to the fungicide had lower body mass (-11 mg) than those from the control group (Table 2, Fig. 5). We found the main effects of fungicide exposure and parental genotype on the male body mass instead of the interactive effect. Fungicide exposed males had significantly lower body mass at all time points (− 12, − 14, and − 11 mg) than those from the control group (Table 2; Fig. 5). In addition, males descending from RR parents had higher emergence (+ 9 mg) and higher after winter (+ 9 mg) body mass than the males from RS parents (Table 2; Fig. 5).

### Fungicide and parental genotype effects on reproduction (i.e., egg production and hatching)

**Figure 6:**
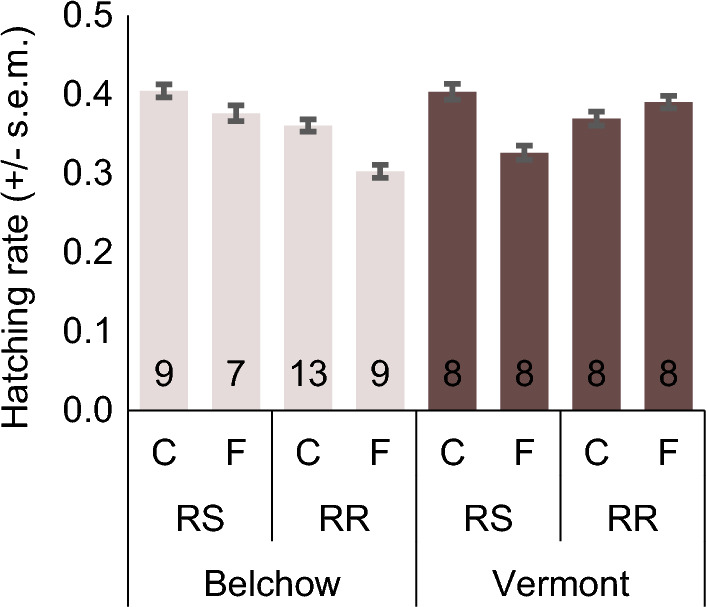
Egg hatching rate (+ / − s.e.m.) in Belchow and Vermont population. RS marks the mixed and RR resistant families. C marks for control and F for fluazinam fungicide treatment. Numbers in the column base show the number of families.

Fungicide exposure, parental genotype, or their interaction did not affect egg production in the investigated populations (Table 3). Nevertheless, we identified a significant fungicide-parental genotype interaction effect on egg hatching in both populations (Table 3; Fig. 6). In the Belchow population, RR individuals exposed to the fungicide laid eggs with a 6% lower hatching rate and individuals from RS families with 3% lower hatching rate than the control group (Table 3; Fig. 6). In the Vermont population, parental fungicide exposure increased egg hatching by 2% among the RR families, while it decreased by 8% among RS families compared to the control group (Table 3; Fig. 6). These results suggest that fungicide exposure effects depend on the parental genotype and can have minor adverse effects on reproduction and thus affect long-term population dynamics.

**Table 3:** Parental fungicide exposure, parental insecticide resistance genotype (RR/RS), and their interaction do not affect egg production but affect egg hatching within 30 days, in the Colorado potato beetles, from Belchow and Vermont populations. Parents were either homozygous (RR) or heterozygous (RS) for the S291G mutation in the *acetylcholinesterase*-*2* gene. Significant results (p < 0.05) are shown in bold.

Measured trait	Population	Model	Wald χ2	p
Egg production	Belchow	parental genotype	0.05	0.824
		parental fungicide exposure	0.005	0.943
		parental genotype* parental fungicide exposure	0.16	0.689
		egg batches	**9.97**	**0.002**
		maternal after winter weight	0.26	0.608
	Vermont	parental genotype	0.02	0.904
		parental fungicide exposure	0.07	0.788
		parental genotype* parental fungicide exposure	0.09	0.763
		egg batches	0.93	0.335
		maternal after winter weight	0.20	0.659
Egg hatching	Belchow	parental genotype	**51.45**	** < 0.001**
		parental fungicide exposure	**28.76**	** < 0.001**
		parental genotype* parental fungicide exposure	**3.89**	**0.049**
		egg batches	**5.17**	**0.023**
		maternal body mass	3.76	0.052
	Vermont	parental genotype	3.00	0.083
		parental fungicide exposure	**11.22**	** < 0.001**
		parental genotype* parental fungicide exposure	**25.57**	** < 0.001**
		egg batches	**5.52**	**0.019**
		maternal body mass	**9.48**	**0.002**

Parents were either homozygous (RR) or heterozygous (RS) for the S291G mutation in the *acetylcholinesterase*-*2* gene. Significant results (p < 0.05) are shown in bold.

## Discussion

Fungicides are the most used pesticides in the European Union^1^ and European food production depends on them. Nevertheless, their ecological effects have been overlooked compared to other pesticides^6^. Here we show that fluazinam-based fungicide exposure has long-term and transgenerational consequences on two different Colorado potato beetle populations and that these effects depend on the individuals’ parental insecticide resistance background. We found that larval fungicide exposure does not cause immediate effects on survival but has long-term life-history consequences on survival at the adult stage, adult body mass, and even on reproduction (egg hatching), depending on population and parental insecticide resistance status. In the Belchow population, we found that fluazinam exposure led to higher larva-to-adult mortality yet caused more resistant individuals to survive better with costs on reducing their offspring quality (egg hatching). However, fungicide exposure did not reduce the survival in the Vermont population but had parental genotype-specific effects on the adult body mass and reproduction. In other words, females descending from RR parents benefitted from the fungicide exposure: they were bigger and produced eggs with higher hatching rates than those not exposed. Females descending from RS parents suffered from fungicide exposure: they had lower adult body mass and produced eggs with lower hatching rates. We found similarly to previous studies, that fungicide exposure does not result in short term lethal effects, but importantly when we studied the effects further, we found instead of commonly found negative effects^7,8^ also some positive effects, depending on the individuals’ parental insecticide background and population.

Previous studies have found that fungicides are considered either harmless based on short-term toxicity tests but can have harmful delayed effects on insects such as cherry-oat aphid (*Rhopalosiphum padi* L.)^8^ and honeybees (*Apis mellifera* L.)^7,11,28^. Therefore, the idea that fungicide exposure can have positive fitness effects on the insect population is intriguing. In our study, positive effects were most apparent in the individuals exposed to fungicide and descended from RR parents. Since the Colorado potato beetle is known to be very resistant to different pesticides^29–31^ it can lead to a phenomenon called cross-resistance where resistance to insecticides can increase tolerance to other pesticides/xenobiotics such as fungicides^20^. Alternatively, positive effects could be induced by the hormetic effects of fungicides. Previous studies on the Colorado potato beetle have identified that fungicides induce similar genetic detoxification mechanisms as insecticides^10,15^. For example, both chlorothalonil and boscalid based fungicide exposure induced phenotypic, enzymatic, and expressional responses in the Colorado potato beetle, which correlated with known mechanisms of insecticide resistance^15^. We have found earlier that exposure to fluazinam-based fungicide (Shirlan) downregulated the *uridine diphosphate glycoronosyl-transferase 1 (UDP)* gene^10^, which is associated with the metabolic detoxification of insecticides^15,32^. It also upregulated the expression of the *acetylcholinesterase-1* gene which is target site of the carbamate and organophosphate insecticides^22^ and the activity of cytochrome P450 which is a common enzyme group involved in metabolic detoxification^33,34^. Hormetic effects stimulated by insecticides^35^ and their possible mechanisms have been reviewed widely^36^. Yet, whether changes in gene expression could lead also to positive transgenerational effects of fungicides and its possible interactive effects with insecticides, should be confirmed in future studies in other organisms using several populations and resistant backgrounds. Our results showing that fungicide exposure can have positive fitness effects and select for insecticide resistance in a pest insect are worrying. Positive effects can give rise to pest outbreaks and contribute to the pesticide treadmill. This could partly explain why Brevik et al.^30^ found that the Colorado potato beetle can evolve resistance nowadays in less than 20 generations, while in 1910 it still took 120 generations.

We also found that the two investigated populations differed in their response to the fungicides. The positive effects in the Belchow could stem from the selection by fungicides on the larva-to-adult survival, while the positive effects in the Vermont population possibly stem from the hormetic effects of the fungicides. This could be because the role of this single mutation differs within the two populations. In Belchow single mutation is likely very important in tolerating the insecticides, while in Vermont, it is linked to other mutations that might play more important role^22^. The fact that populations differ is not surprising, yet these effects are often not studied. Population level differences could depend on several factors such as insecticide resistance background, fungicide exposure history, geographical history, as well as genetic or fitness level differences between populations. We suggest that the population level differences come mainly from the differences in the insecticide resistance background as like we discussed before, fungicides have been reported to induce insecticide like effects on this species^10,15^ and hence it is likely that more resistant population is less sensitive. Based on previous studies, these populations are known to differ significantly in their resistance to organophosphate and carbamate insecticides^22^ and glyphosate-based herbicide^37^. Alternatively, population level differences could be also caused by the differences in the genetic variability^26,38^. Higher genetic variability could contribute to the adaptation to different pesticides. Indeed, we show that the less variable Belchow population^38^ is more sensitive to the fungicide exposure, which could be due to disability to adapt to exposures. Here, we think that fungicide application history is not likely affecting the population level difference because fungicides are used more in the European countries than in the USA^1,6^.

Our study is not without limitations, first, we were only able to compare individuals that were either homozygous resistant (RR) or heterozygous (RS) for the S291G mutation, and we were lacking the homozygous susceptible (SS) individuals from the study. Based on the current study (Fig. 2) as well as earlier studies, the susceptible individuals are very rare in these particular populations, but also in other beetle populations^22,38^. Moreover, even though we are missing the susceptible individuals from the mated pairs, we think that our study is therefore more realistic, as the used genotypes are also more common in the agricultural fields. Second, we conducted the study in laboratory, which allowed us to clearly identify the fungicide effects, as all the other factors were the same for two treatment groups and populations. However, in future, these positive effects that we found in the laboratory conditions should be tested in the field conditions because they can interact in a synergistic or antagonistic way with other variables, such as climate or insecticide application. Finally, we treated the beetles with fungicides only once during the larval stage, while in the field Shirlan fungicide can be applied up to 8 times according to the manufacturers’ recommendations with 7- to 14-day intervals and the short interval (*i.e.,* 7 days) is recommended only when the blight is widely spread in the field. Therefore, the long-term effects of multiple exposure could be studied further. Though, for the beetle these might not be that relevant, as its development time from egg to larva under lab conditions is *ca* 11–14 days (Margus, personal observations), which suggests that they are likely exposed to the Shirlan fungicide only once during the larval period.

## Conclusions

Taken together, our findings demonstrate that fungicide exposure can have both negative and positive long-lasting effects on the non-target insect species, depending on population and the presence of insecticide resistance mutation, underlining that the effects of fungicide on non-target species may be difficult to predict. Negative effects of fungicide exposure include increased larva-to-adult mortality and reduced size in individuals from RS parents. Thus, the fungicide exposure may inadvertently lead to higher frequency of insecticide resistance at the population level. Conversely, we also observed positive long-term effects, such as increased body mass in females descending from RR parents. Heavier individuals in turn survive better during winter and usually produce more offspring. The positive transgenerational effects of fungicide treatment on the fitness of a pest species could contribute to the pest outbreaks and explain pesticide treadmill and hence all agrochemicals should be considered together in the management strategies.

## Materials and methods

### Study species

We used the laboratory population of the Colorado potato beetles initially collected from Vermont (44° 43′ N, 73°20′ W), USA and Belchow (52° 01′ N, 20° 34′ E), Poland, in 2010. Since then, the beetles have been mated and reared under laboratory conditions using controlled climate chambers (detailed rearing conditions described in^39,40^. We conducted the experiments in the summer of 2020 after the 9th beetle generation. As the main pest of potato, the beetle is the most common non-target pest of fungicides used in the potato field to control potato blight making it an excellent species to study fungicide effects. For example, early blight (*Alternaria solani* Soraurer) and late blight (*Phytophthora infestans* (Mont.) de Bary) diseases can be treated with fungicides approximately seven times during the summer, while the beetle is treated with insecticides only three times annually^41^.

### Genotyping adults for S291G mutation in the *acetylcholinesterase-2* gene

At the beginning of the experiment, we determined the beetles’ insecticide resistance status. We genotyped individuals for S291G mutation in the *acetylchonesterase-2* gene, which is associated with resistance to organophosphate and carbamate insecticides^24,27^. First, we extracted DNA from the hindwing with Qiagen DNeasy (Qiagen, Germany) tissue kit reagents and a Kingfisher magnetic particle processor. Then we Sanger sequenced the mutation using primers obtained from Clark et al.^27^. Next, we determined the resistance status of the beetles based on the non-synonymous serine to a glycine point mutation at site 291 (i.e. S291G)^27^. Based on the mutation, individuals were either homozygous resistant (RR), heterozygous resistant (RS), or homozygous susceptible (SS) if they lacked the mutation (Fig. 2).

### Experimental setup and fungicide exposure

After genotyping, we randomly mated the females with unrelated males within the population based on their resistance status (see experimental setup Fig. 1). The RR group contained families where both female and male beetles were homozygous resistant for the S291G mutation. Due to the lack of susceptible individuals and a low number of heterozygote individuals (Fig. 2), in the RS group families, we mated heterozygous beetles with homozygous resistant beetles. We observed the mated pairs, fed the adults, and collected their eggs every other day. Egg hatching was checked daily. We exposed the larvae from each family to fungicide or control treatment. As a fungicide treatment, we used the highest field-related concentration of fluazinam-based fungicide Shirlan (Syngenta Crop Protection AG, Switzerland) that is recommended by the manufacturer in Finland (0.4 l/ha), and dH_2_O as control^10^. We divided 20 to 30 larvae from the same family into control and fungicide treatment. For the exposure, we pipetted 1 µl of the fungicide solution (0.66 mg/l of fluazinam) or water on the back of the larva. Each larva was exposed once to the fungicide, kept on Petri dishes for 72 h to check their survival, and fed daily with fresh potato leaves (variety Challenger). After 72 h of exposure, we transferred the larvae to the fully-grown potato plants to allow them to feed and pupate in the soil. We placed the potato plants in the controlled growth chamber (FH-1300, HiPoint, Taiwan) using constant 23 °C and long day conditions of 18 h of light (with 2 h of dim light) and 6 h of dark. After pupation, we recorded larva-to-adult survival and measured their body mass with a scale (AM100, Mettler, Columbus, OH, USA). Newly emerged adults were transferred into short day conditions of 12 h of light (with 2 h of dim light) and 12 h of dark to induce winter diapause^42^. Each newly emerged adult was placed on a Petri dish separately and fed *ab libitum* with potato leaves until diapause. After ten days of feeding, we weighed the individuals again and moved them to plastic jars containing soil for overwintering. Jars were transferred to 23, 20, 15, and 10 °C with two-week intervals and finally kept at 5 °C, in constant darkness during the winter for ca 9 months.

In the following spring, we increased the temperature gradually by five degrees, from an initial 5 °C to a final 23 °C, in two-weeks intervals to induce diapause termination in the beetles in June. Once beetles emerged from the soil, we weighed and moved them to Petri dishes, where they were fed ad libitum with fresh potato leaves and stems. Beetles were kept at 23 °C in long-day conditions to allow them to regain their fat reserves and were genotyped for the S291G mutation during 23–49 days, after which we mated the second generation. We genotyped the individuals only from the RS genotype families. The second-generation families consisted of females randomly mated with unrelated males within the population: RR group consisted of families where both female and male were homozygous resistant for the S291G mutation, and the RS group (see sample sizes in Fig. 2) consisted of families where males were heterozygous and females homozygous resistant because of the low numbers of heterozygous females. We observed mated pairs every other day, fed the adults, and collected their eggs. To study the effects of fungicide exposure on reproduction, we counted the number of eggs and hatched larvae within 30 days of the first egg-laying date.

### Statistical analysis

We analysed the data with the IBM SPSS Statistics version 26.0 (Armonk, NY, IBM Corp) program for two populations separately. Survival (dead/alive) was analysed with a binary logistic generalised linear model. Survival at different life stages was set as a dependent variable, and fungicide treatment, genotype, and their interaction were set as predicting factors. Differences in body mass (mg) was analysed for two population and sexes separately with ANCOVA, where fungicide treatment and genotype were set as fixed variables and family was included as a covariate. Egg production (number of eggs) was analysed with the negative-binomial regression model, and egg hatching (hatched/did not hatch) was analysed with the binary logistic regression model. We used the same explanatory variables for both responses: fungicide treatment, parental genotype, and their interactions as fixed factors and after winter weight of the mother and number of egg batches as covariates. See the descriptive characteristics of the study groups (Supplementaty Table 1).

### Supplementary Information

Supplementary Table 1.

## Data Availability

The datasets analysed in the current study are available in the JYX Digital Repository (https://jyx.jyu.fi/).
