# Supplementary material for: Fungicides modify pest insect fitness depending on their genotype and population
Source: Sci Rep. 2023 Oct 19;13:17879. doi: 10.1038/s41598-023-44838-5 (PMC10587347; doi:10.1038/s41598-023-44838-5)
Supplement: Supplementary file 1 — Supplementary Table 1. [file 41598_2023_44838_MOESM1_ESM.docx]

**FUNGICIDES MODIFY PEST INSECT FITNESS DEPENDING ON THEIR GENOTYPE AND POPULATION.**

Aigi Margus^1*^, Shahed Saifullah^1^, Maaria Kankare^1^, and Leena Lindström^1^

^1^Department of Biological and Environmental Science, P.O. Box 35, University of Jyväskylä, FI-40014 Finland

*Corresponding author

ORCID ID-s: AM 0000-0002-2546-8238, SS 0009-0007-7276-1641, MK 0000-0003-1541-9050, LL 0000-0002-6452-4538.

**Supplementary table 1.** Descriptive characteristics of the study groups (mean ± s.e.m.) for the Colorado potato beetles from Belchow and Vermont populations, mixed (RS + RR) and resistant (RR + RR) families, and control and fungicide exposure groups.

|  | Belchow | | | | Vermont | | | |
| --- | --- | --- | --- | --- | --- | --- | --- | --- |
|  | RS + RR | | RR + RR | | RS + RR | | RR + RR | |
|  | Control | Fungicide | Control | Fungicide | Control | Fungicide | Control | Fungicide |
| *Survival* |  |  |  |  |  |  |  |  |
| 72- hour (%) | 100 ± 0 | 99 ± 1 | 99 ± 1 | 99 ± 1 | 98 ± 2 | 100 ± 0 | 100 ± 0 | 97 ± 2 |
| Larva to adult (%) | 74 ± 5 | 56 ± 6 | 61 ± 5 | 44 ± 5 | 71 ± 6 | 69 ± 6 | 80 ± 4 | 72 ± 5 |
| Before diapause (%) | 100 ± 0 | 92 ± 4 | 98 ± 2 | 97 ± 3 | 98 ± 2 | 98 ± 2 | 98 ± 2 | 96 ± 2 |
| Winter survival (%) | 87 ± 5 | 81 ± 7 | 82 ± 5 | 97 ± 3 | 87 ± 5 | 93 ± 4 | 90 ± 4 | 94 ± 3 |
| *Female body mass* |  |  |  |  |  |  |  |  |
| Emergence (mg) | 128 ± 4 | 110 ± 5 | 131 ± 4 | 138 ± 5 | 124 ± 4 | 113 ± 4 | 125 ± 4 | 131 ± 4 |
| Before diapause (mg) | 169 ± 5 | 150 ± 7 | 180 ± 5 | 180 ± 6 | 157 ± 5 | 140 ± 5 | 164 ± 4 | 171 ± 5 |
| After winter (mg) | 155 ± 5 | 142 ± 6 | 158 ± 5 | 158 ± 5 | 145 ± 4 | 131 ± 4 | 146 ± 4 | 156 ± 4 |
| *Male body mass* |  |  |  |  |  |  |  |  |
| Emergence (mg) | 106 ± 4 | 106 ± 4 | 109 ± 3 | 115 ± 4 | 113 ± 4 | 97 ± 4 | 118 ± 3 | 110 ± 3 |
| Before diapause (mg) | 147 ± 5 | 145 ± 5 | 150 ± 4 | 152 ± 6 | 144 ± 5 | 131 ± 5 | 151 ± 4 | 134 ± 4 |
| After winter (mg) | 131 ± 4 | 123 ± 5 | 133 ± 4 | 135 ± 5 | 127 ± 5 | 115 ± 4 | 135 ± 3 | 125 ± 4 |
| *Reproduction* |  |  |  |  |  |  |  |  |
| Egg production (nr) | 377 ± 130 | 306 ± 86 | 322 ± 122 | 342 ± 115 | 489 ± 216 | 452 ± 180 | 389 ± 150 | 465 ± 165 |
| Egg hatching rate (%) | 40 ± 0.8 | 38 ± 1.0 | 36 ± 0.8 | 30 ± 0.8 | 40 ± 1.0 | 33 ± 0.9 | 37 ± 0.9 | 39 ± 0.8 |
